# Efficacy of two siddha polyherbal decoctions, Nilavembu Kudineer and Kaba Sura Kudineer, along with standard allopathy treatment in the management of mild to moderate symptomatic COVID-19 patients—a double-blind, placebo-controlled, clinical trial

**DOI:** 10.1186/s13063-021-05478-0

**Published:** 2021-08-28

**Authors:** Anurag Srivastava, Manickavasagam Rengaraju, Saurabh Srivastava, Vimal Narayanan, Vivek Gupta, Rashmi Upadhayay, Jitender Kumar, Sathiyarajeswaran Parameswaran, KanakavalliKadarkarai, AarthiVelmurugan

**Affiliations:** 1 Government Institute of Medical Sciences (GIMS), Greater Noida, India; 2 Siddha Clinical Research Unit, Safdarjung Hospital, New Delhi, India; 3 Centre for Computational Biology and Bioinformatics, Amity University Uttar Pradesh, Noida, India; 4 Siddha Central Research Institute, Chennai, India; 5 Central Council for Research in Siddha, Chennai, India

**Keywords:** Mild to moderate COVID-19, Siddha medicine, Kaba Sura Kudineer, Nilavembu Kudineer, Double-blinded RCT

## Abstract

**Background and aim:**

Globally, the ongoing pursuit in exploring an effective drug to combat severe acute respiratory syndrome coronavirus-2 (SARS-CoV-2) virus has not met with significant success to date. Indian traditional medicines, especially polyherbal formulations like Nilavembu Kudineer (NVK) and Kaba Sura Kudineer (KSK) of the Siddha system of medicine, have been used as public health interventions for controlling viral epidemics like dengue and Chikungunya. These traditional therapies have been found safe, effective, and widely accepted. The current study evaluates the comparative efficacy of NVK and KSK as opposed to the placebo, in the management of mild to moderate COVID-19 disease.

**Methods:**

The study was a double-blind, placebo-controlled comparative clinical trial, with the primary objective of determining the efficacy of KSK and NVK. Patients (*n*=125) diagnosed with mild to moderate COVID-19 symptoms were enrolled in the study over a period of 4 months (Aug 2020—Dec 2020). Participants were randomized into 3 arms; placebo-decaffeinated tea in Arm I, NVK in Arm II, and KSK in Arm III. Each arm received 60 ml of the respective treatment twice a day, post morning and evening meals, along with standard allopathy treatment for a maximum of 10 days. The main outcome measures of the study were the reduction in SARS-CoV-2 viral load, hospital stay, and time taken by the patients to become asymptomatic from symptomatic. Efficacy assessments included clinical symptoms (fever, cough, and breathlessness) each day and real-time reverse transcription-polymerase chain reaction (RT-PCR), liver function test (LFT), renal function test (RFT), and electrolytes and electrocardiogram (ECG) at baseline (day 0) and days 3, 6, and 10. Post-treatment, participants were followed up for 30 days via phone for adverse effects if any. Effects of drugs on inflammatory markers (IL6) at the end of treatment were also recorded. Adverse events (AE) were monitored throughout the study.

**Results:**

The results revealed that when compared to patients in the placebo arm, those in NVK and KSK arms showed a statistically significant reduction in hospital stay time, reduction in viral load of SARS-CoV-2, and the time taken to become symptomatic from asymptomatic. Out of 125 COVID-19 patients recruited, 120 completed the study; two from the placebo group developed severe symptoms and were shifted to the intensive care unit (ICU) and three patients from Arms II and III withdrew from the study. The mean age of females (*n*=60) and males (*n*=60) enrolled was between 40.2 and 44.3 years, respectively. Results were more promising for all the patients in NVK and KSK arms as all enrolled participants (100%) under this group got discharged by day 6 as compared to only 42.5% (*n*=17) from the placebo group on that day. The hospital stay time for patients in Arm I was significantly longer (mean [SD]=8.4 [2.0] days) as compared to the Arms II and III (mean [SD]=4.7 [1.5] and 4.2 [1.5] days, respectively (Kruskal-Wallis test, *P*=0.0001). Patients in the three groups took a significantly different number of days to become asymptomatic. While Arm II and III patients took mean of 2.5 and 1.7 days, respectively, Arm I, patients took a mean of 4.2 days (Kruskal-Wallis test, *P*=0.0001). In all, two adverse events were recorded, one for vomiting and one for diarrhea lasting a day in Arm I and Arm II, respectively. The mean value of interleukin-6 (IL6) was significantly different in comparison to the placebo-decaffeinated tea arm (NVK=2.6 and KSK=2.2, placebo=4.0, *P*=0.02). The other blood biochemical parameters like C-reactive protein (CRP), lactate dehydrogenase (LDH), ferritin, and D-dimer that were analyzed at the baseline and at the time of discharge from the hospital, were not significantly different in the three arms.

**Conclusion:**

NVK and KSK arms showed a statistically significant reduction in hospital stay time, reduction in viral load of SARS-CoV-2, and time taken for patients to become asymptomatic from symptomatic, when compared to the placebo (decaffeinated tea). The primary outcome measures of the KSK arm were significantly better than those in the NVK arm.

## Introduction

Globally, there has been an ongoing pursuit in exploring an effective treatment to combat severe acute respiratory syndrome coronavirus 2 (SARS-CoV-2). However, this quest across various treatment verticals has led to despair amongst the scientific community [1]. In India, the role of traditional treatments especially Siddha medicines in the management of various diseases is well known that has proven effective, safe, and widely accepted across all ages. During the chikungunya and dengue epidemic in the year 2015 in Tamilnadu, India, the administration of Nilavembu Kudineer (NVK) played a major role in controlling the morbidity [2]. Siddha medicine has contributed to lowering the disease burden during public health emergencies. These medicines could be repurposed for the management of COVID-19. However, there is limited evidence for the integrative treatment approach (standard of care, allopathy treatment along with Siddha medication) in the management of COVID-19.

COVID-19 is a respiratory tract infection caused by a newly emergent coronavirus, SARS-CoV-2, that was first reported in December 2019. At present, we have limited evidence from randomized clinical trials to support pharmacological treatments from conventional medicine for COVID-19 [3].

According to Siddha Medical Literature, the symptoms and signs of COVID-19 including cold, cough, and fever are analogous to Kaba Suram [4, 5].Standard Siddha medicines for tackling these conditions are Kabasurakudineer (KSK) and Nilavembu Kudineer (NVK). NVK was one of the essential medicines used as anti-viral Siddha drugs, especially in the treatment of chikungunya and dengue during the past outbreaks [6]. Recent in vitro studies have revealed that ethanolic extract of NVK has anti-viral properties against chikungunya and dengue [2, 7]. Toxicity studies utilizing NVK as per Organisation for Economic Co-operation and Development (OECD) guidelines found it to be safe for consumption. Apart from this, antipyretic, anti-microbial, anti-inflammatory, and immunostimulant activities of NVK have also been proven by phytochemical screening studies [8]. Recent clinical studies have revealed the prophylactic and antiviral activities of NVK in viral fevers [9, 10]. These indicate the growth inhibition of viral pathogens, and the ability to effectively inhibit spill-over and transmissibility of the viruses. Therefore, in the current study, NVK was selected as one of the drugs against COVID-19.

KSK is a classical Siddha formulation comprising of 15 herbs and each herb possesses antiviral activity [11, 12]. It has been found that a few phytocomponents in KSK decoction such as Cucurbitacin B (-112.09), Cardiofoliolide (-111.5), Apigenin (−98.84), and Pyrethrin (−92.98) bind to the virus and inhibits its replication hence could be effective in the management COVID-19. In silico studies of KSK ingredients have shown to be potent against SARS-CoV-2 spike proteins [13]. Determination of organoleptic characters, preliminary phytochemical analysis, physicochemical analysis, thin layer chromatography (TLC) photodocumentation, and high-performance thin-layer chromatography (HPTLC) fingerprint studies on KSK are reported [14]. A study has shown that KSK has antipyretic, anti-inflammatory, and anti-bacterial properties and found to be safe in toxicity test [15]. KSK has also been shown as an immuno-modulator and having thrombolytic activity [16]. A retrospective observational study to measure the effect of integrated therapy KSK with vitamin C and zinc on COVID-19 patients has proven that there is a reduction in length of hospital stay [17, 18].

In the absence of a systematic evaluation of integrated therapy (with the standard of care, allopathy and KSK, or NVK from Siddha system of medicine in COVID-19 management), this was proposed as a comparative study.

## Materials and methods

The study was conducted at the Government Institute of Medical Sciences (GIMS), Greater Noida, Uttar Pradesh, India. Patients were enrolled from August 22, 2020, to December 31, 2020. The Ethics Committees of the participating site and Siddha Clinical Research Unit, New Delhi, Safdarjung Hospital, approved the protocol. Prior to participation in the study, each patient was informed about the nature and purpose of the study and written informed consent was obtained. All research procedures were strictly adhered to, based on AYUSH GCP and Indian Council for Medical Research (ICMR) Guidelines. The trial was registered in the Clinical Trial Registry of India (CTRI), and the registration number is CTRI/2020/08/027286 [19]. The detailed protocol of the study was already published [19].

### Study design

**Fig. 1:**
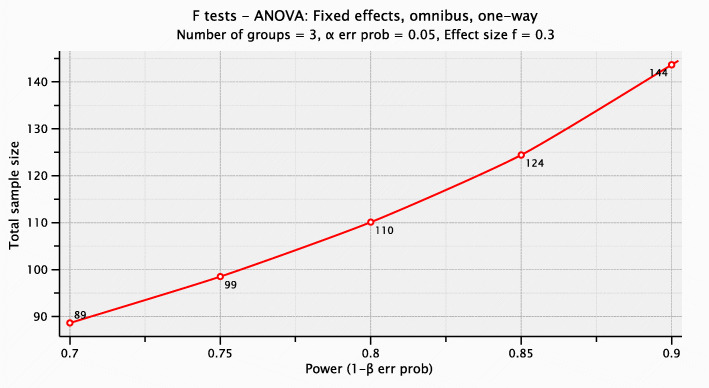
Sample Size Calculation

**Fig. 2:**
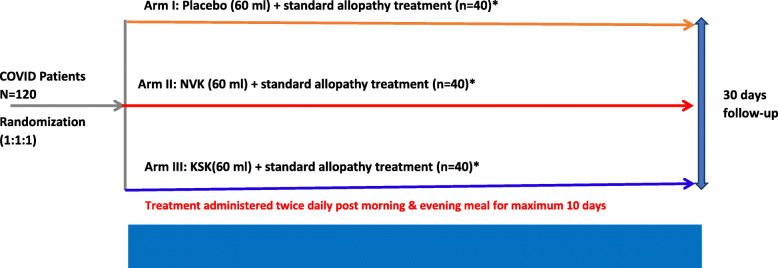
Study design displaying treatment allocation for each arm. *Standard allopathy treatment: doxycycline/hydroxychloroquine and Ivermectin/Fabiparavir and patients with moderate disease also received steroids (methyl prednisolone/dexamethasone)and low molecular weight heparin. ^#^Clinical symptom assessment for fever, breathlessness, respiratory rate, oxygen saturation, and cough were recoded daily

This was a randomized, double-blind, placebo-controlled, clinical trial where mild/moderate patients were randomly assigned to study treatment in a 1:1:1 ratio, to placebo (Arm I) or NVK (Arm II) or KSK (Arm III) groups. Patient allocation to the treatment arm was performed using a simple stratified randomization method. The sample size was determined based on calculation for effect size 0.30, and the total sample size in the three groups is coming out to be 110 (Fig. 1). Considering a dropout rate of 10%, we have to recruit 125 patients for the study. Please find in Fig. 2 for more clarity.

#### Eligibility criteria

Eligible patients were 18–65 years of age with mild to moderate symptoms of COVID-19 and willing to consent. COVID-19 was confirmed by RT-PCR screening following the ICMR guidelines. A total of 155 confirmed COVID-19 patients with mild to moderate symptoms of COVID-19 were screened at the site. 125 patients were enrolled and admitted to the hospital, and all the guidelines laid by ICMR, Govt. of India, for COVID-19 management were followed. The participants were categorized as mild or moderate COVID-19 following WHO criteria [20]. The patients having oxygen saturation (SpO_2_) of <94%>90%, respiratory rate >24 breaths per minute, and chest X-ray showing pneumonia were classified as moderate COVID-19 patients, whereas patients with SpO_2_>94% and respiratory rate <24 breaths per minute were classified as mild COVID-19 patients. Patients were excluded from the study if they had a severe primary respiratory disease or other pathogenic microbial pneumonia, with uncontrolled diabetes mellitus (≥350 mg/dL fasting sugar), severe hypertension (HT) (180/120 mmHg), chronic bronchial asthma (BA) (≥5 years based on clinical history), renal dysfunction (known chronic kidney disease [CKD] ≥5 years estimated glomerular filtration rate (eGFR) stage ≥3), and pregnant and lactating mothers. Patients with other systemic malignant diseases such as malignant tumors, mental illnesses, which the researchers considered unsuitable for participation in the study, people who had a history of allergy to Siddha medicine or who were part of other COVID-19 clinical trials were excluded from the study.

### Study treatment

All the patients in the three arms received standard allopathy treatment as per ICMR guidelines, which included doxycycline/hydroxychloroquine and Ivermectin/Fabiparavir. Additionally, the patients with a moderate disease also received steroids (methylprednisolone or dexamethasone, if required) and low molecular weight heparin. Participants were randomized to receive 60 ml of placebo in Arm I, 60 ml of NVK in Arm II, and 60 ml of KSK in Arm III, twice a day post morning and evening meals along with standard allopathy treatment, for a maximum of 10 days. Post-treatment, patients were followed up for 30 days via phone for safety. The study design is displayed in Fig. 2.

### Procedure to prepare a polyherbal decoction of KSK and NVK

Both the KSK and NVK decoctions were prepared as per the Siddha Formulary of India Guidelines [21]. In order to obtain NVK or KSK decoctions, a 5-mg coarse powder of NVK or KSK, obtained from the Central Pharmacy-Central Council for Research in Siddha (CCRS), Chennai, India, was boiled in 240 ml of water and reduced to one fourth (60ml), followed by filtration. The composition of polyherbal decoction ingredients of both NVK and KSK are detailed in Tables 1 and 2, respectively.

**Table 1:** Composition and polyherbal decoction ingredients of Nilavembu Kudineer (NVK) as per Siddha Formulary of India Guidelines [21]

S. No	Botanical name	Siddha name	Family	Part used	Parts
1	*Andrographis paniculata (Burm.f.)*	Nilavembu	Acanthaceae	Whole plant	1 part
2	*Vetiveria zizanioides* L.	Vettiver	Poaceae	Whole plant	1 part
3	*Santalum album* L.	Santhanam	Santalaceae	Wood	1 part
4	*Zingiber officinale Roscoe.*	Chukku	Zingiberaceae	Rhizome	1 part
5	*Piper nigrum L.*	Milaku	Piperaceae	Dry fruits	1 part
6	*Cyperus rotun*dus *L.*	Korai kilanku	Cyperaceae	Rhizome	1 part
7	*Hedyotis corymbosa L.Lam*	Parpadagam	Convolvulaceae	Whole plant	1 part
8	*Plectranthus vettiveroides (K.C.Jacob) N.P.Singh & B.D.Sharma*	Vilamicham ver	Lamiaceae	Root	1 part
9	*Trichochanthes cucumerina* L.	Peipudal	Cucurbitaceae	Whole plant	1 part

**Table 2:** Composition and polyherbal decoction ingredients of Kaba Sura Kudineer (KSK)) as per Siddha Formulary of India Guidelines [21]

S. no	Botanical name	Siddha name	Family	Part used	Parts
1	*Zingiber officinale Roscoe.*	Chukku	Zingiberaceae	Rhizome	1 part
2	*Piper longum L.*	Milagu	Piperaceae	Fruit	1 part
3	*Syzygium aromaticum (L,) &L.M Perry*	Kirambu	Myrtaceae	Flower bud	1 part
4	*Anacyclus pyrethrum L.*	Akkarakaram	Asteraceae	Rhizome	1 part
5	*Tragia involucrate L.*	Siru kanjori	Euphorbiaceae	Leaves	1 part
6	*Solanumanguivi Lam*	Karimulli	Solanaceae	Leaves	1 part
7	*Terminalia chebula (Gaertn.)*	Kadukkai	Combretaceae	Fruit rind	1 part
8	*Justicia adathoda* Linn.	Adathoda	Acanthaceae	Leaves	1 part
9	*Anisochilus carnosus* (*L.f) Wall, ex Benth*	Karpoora valli	Lamiaceae	Whole plant	1 part
10	*Costus speciosus (J.Koing)Sm*	Koshtam	Costaceae	Rhizome	1 part
11	*Tinospora cordifolia (Thunb.) Miers,*	Seenthil	Menispermaceae	Whole plant	1 part
12	*Clerodendrum serratum (L.)*	Siru Theku	Verbanaceae	Leaves	1 part
13	*Andrographis paniculata (Burm.f.)*	Nilavembu	Acanthaceae	Whole plant	1 part
14	*Cyperus rotundus L.*	Korai Kilanku	Cyperaceae	Rhizome	1 part
15	*Sida acuta (Burm.f.)*	Sitramutti	Malvaceae	Whole plant	1 part

**Procurement and preparation:** NVK and KSK were procured from the GMP certified pharmacy (Central Pharmacy – CCRS, Chennai). A 5-mg coarse powder of NVK or KSK was boiled in 240 ml of water and reduced to one fourth (60ml), followed by filtration, to obtain NVK or KSK decoctions

**Fig. 3:**
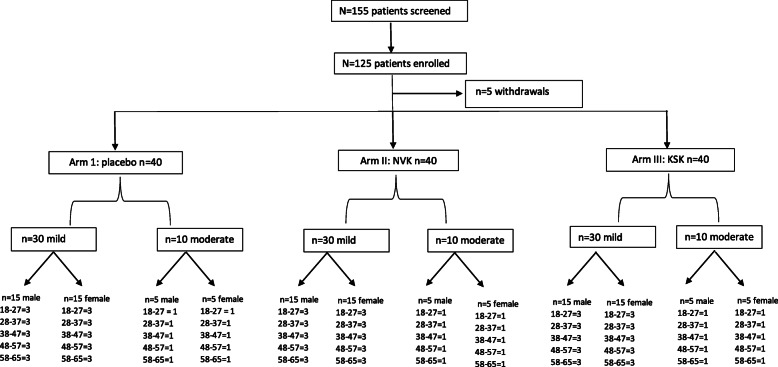
Subject disposition displaying screening and treatment allocation of participants. A total 125 patients were enrolled, and 5 patients withdrew from study. Out of 120 patients, all were equally allotted into 3 groups each 40. In that 40, further divided 3:1 ratio for mild and moderate cases (based on ICMR, Min. of Health COVID-19 Criteria). Here, male/female patients were also equally distributed to all three groups. Randomization done by simple stratified randomization

The distribution of participants included in the study (*N*=125) is summarized in Fig. 3. Enrolment of 120 subjects was planned. However, a total of 125 patients were enrolled (Arm 1: 42, Arm 2: 43, and Arm 3: 40) as 5 patients withdrew (Arm 1: 2 and Arm 2: 3) before the start of treatment. A total of 120 patients completed the study; 40 patients each in Arms 1, 2, and 3. In each arm, the male (*n*=20) and female (*n*=20) patients were equally distributed by stratified randomization. In each arm, the 40 patients were further divided into 3:1 ratio for mild (*n*=30) and moderate (*n*=10) cases based on ICMR, the Ministry of Health COVID-19 Criteria.

### Outcome measures

The primary outcome measures were the reduction in SARS-CoV-2 viral load (RT PCR Ct value), time taken by the patient to become asymptomatic from symptomatic, and reduction in the hospital stay. The patient was discharged from the hospital if they were RT PCR negative. Immunity markers (IL-6) and other biological and hematological markers (CRP, LDH, D-dimer, and ferritin) at baseline and on the day the patient got discharged were also analyzed.

### Efficacy evaluations

Clinical assessment for symptoms like fever (≤36.6°C or -axilla, ≤37.2°C oral or ≤37.8°C rectal or tympanic, cough), breathlessness (respiratory rate ≤24/minute on room air, oxygen saturation (SpO_2_) >94% on room air, cough -mild or absent on a patient-reported scale (cough symptoms score ≤2 points, SpO_2_ level ≥95 was recorded each day throughout the study. Laboratory assessments including RT-PCR, LFT, RFT, and electrolytes and ECG were performed at baseline (day 0), and day of discharge (days 3, 6, and 10).

### Statistical analysis

Statistical analysis was performed using R Commander for R. The continuous variables were checked for normality tests, and those who did not follow normal distribution were transformed taking square root values. Continuous data was reported as the mean (standard deviation) while categorical data was reported as number (percentage). Three groups were compared for different biochemical parameters using Kruskal-Wallis test. Chi-square test was performed to compare proportions among groups. Values were considered significant if *p* value <0.05.

### Blinding and randomization

Blinding and randomization were employed to avoid bias in the assignment of participants to treatment, to increase the likelihood that known and unknown subject attributes (e.g., demographics and baseline characteristics) were evenly balanced across treatment groups, and to enhance the validity of statistical comparisons across treatment groups. Participants were randomly assigned to either placebo, NVK, and KSK arm by an allocation ratio of 1:1:1. Blinded treatment was used to reduce potential bias during data collection and evaluation of clinical endpoints in the study. Measures were taken to ensure that the study patients and study staff were not unblinded. Study participants and investigators were blinded. Random allocation was done by a statistician who was not involved in the study. Siddha pharmacist who prepared herbal decoctions was not involved in the study, hence was unaware of which patient was getting which decoction. A placebo group was included to have an accurate assessment study treatment.

## Results and discussion

### Study subjects’ demographic characteristics

The demographic and baseline characteristics were comparable across treatment groups. A total of 120 patients completed the study and comprised of equal number males 50% (*n*=60) and females 50% (*n*=60). The mean age of Arm I, Arm II, and Arm III was found out to be 44.4, 42.8, and 39.5 years, respectively, and was statistically insignificant (*P*=0.26).

### Mean hospital stay time: reduction in hospital stay time

The number of patients discharged on days 3, 6, and 10 from Arms I, II, and III are summarized in Table 3. The patient was discharged from the hospital if the RT-PCR test was negative.

**Table 3:** Numbers of patients discharged in Arm I, Arm II, and Arm III on days 3, 6, and 10

Time patient discharged days	Arm I: placebo ***n***=40	Arm II: NVK ***n***=40	Arm III: KSK ***n***=40
**Day 3** **Male (*n*=20)** **Female (*n*=20)**	0 (0%) 0 (0%) 0 (0%)	16 (40%) 10 (50%) 6 (30%)	24 (60%) 13 (65%) 11 (55%)
**Day 6** **Male (*n*=20)** **Female (*n*=20)**	17 (42.5%) 13 (65%) 4 (20%)	24 (60%) 10 (50%) 14 (70%)	16 (40%) 7 (35%) 9 (45%)
**Day 10** **Male (*n*=20)** **Female (*n*=20)**	23 (57.5 %) 7 (35%) 16 (80%)	0 (0%) 0 (0%) 0 (0%)	0 (0%) 0 (0%) 0 (0%)

In Arm I (the placebo arm), no patients recovered from COVID-19 disease on day 3 after admission. On day 6, 65% (*n*=13) male and 20% (*n*=4) of female patients were discharged. The majority of patients, which was 35% (*n*=7) of male and 80% (*n*=16) female were discharged on day 10.

In Arm II (NVK arm), patients recovered faster in comparison to placebo hence got discharged earlier. On day 3, 50% (*n*=10) of male and 30% (*n*=6) of female patients were discharged. Remaining patients of NVK arm were discharged on day 6, i.e., 50% (*n*=10) of male and 70% (*n*=14) female, respectively. Since all patients recovered and discharged by day 6, hence patients discharged on day 10 of the study were zero.

Patients in Arm III (KSK arm) showed faster recovery even when compared to the NVK arm (Arm II). On day 3, the majority of the patients 65% (*n*=13) of males and 55% (*n*=11) of females were discharged on day 3. The remaining patients, 35% of male (*n*=7) and 45% (*n*=9) of female patients were discharged on day 6. Since all patients were discharged by day 6, there were zero discharges on day 10 from Arm III.

In patients in the placebo arm who were on decaffeinated tea along with allopathy treatment, no patients were discharged on day 3. In fact, the majority, i.e., 23 patients (57.5%) were discharged on day 10. Interestingly, all the patients taking NVK and KSK showed early recovery signs and were discharged by day 6. Further, the patients in the KSK arm recovered even faster than the NVK arm as 60% of patients of the KSK arm were discharged on day 3, as compared to only 40% in Arm II (NVK arm) on the same day.

**Fig. 4:**
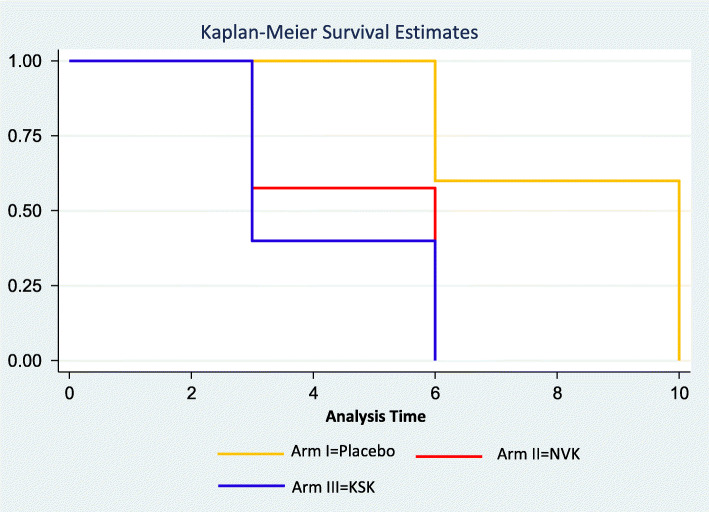
Comparison of Hospital Stay Time Kaplan-Meier graph for Arm I, Arm II, and Arm III. Patients in Arm I stayed significantly longer (mean [SD]=8.4 [2.0]) as compared to the Arms II and III (mean [SD]=4.7 [1.5] and 4.2 [1.5], respectively, Kruskal-Wallis test, *P*=0.0001

The total number of days the patients stayed in the hospital during the treatment was recorded and compared to know if there was a reduction in hospital stay time. Patients in the placebo arm stayed significantly longer (mean [SD] = 8.4 [2.0]) as compared to the Arms II and III (mean [SD]=4.7 [1.5] and 4.2 [1.5], respectively, Kruskal-Wallis test, *P*=0.0001). Comparison of the Hospital Stay Time Kaplan Meier Graph for Arm I, Arm II, and Arm III is presented in Fig. 4. Hence, patients who were taking the Siddha treatment along with allopathy treatment (Arms II and III) had spent almost half the time in comparison to the placebo arm. Overall, the KSK group showed a statistically significant reduction in the hospital stay time compared to standard Siddha drug NVK and placebo which is decaffeinated tea.

### Reduction in viral load of SARS-CoV-2

In order to know the viral load, RT-PCR was performed on days 3, 6, and 10. In Arm I, all patients were RT-PCR positive on day 3, whereas 38% of Arm II and 56% of Arm III got RT-PCR-negative. On day 6, patients tested RT-PCR-negative were 38% (Arm I), 62% (Arm II), and 44% (Arm III), respectively. On day 10, the remaining 61% of patients of Arm I were RT-PCR-negative whereas there were no patients on day 10 in both NVK and KSK arms, since all had got discharged by day 6. In comparison, the KSK treatment arm showed an early reduction in viral load as 56% of patients of this arm were RT-PCR-negative even on day 3 after admission.

Cycle threshold (Ct) values in RT PCR were analyzed at baseline (day 0) and day 3, and results are summarized in Table 4. At baseline, Ct values of three arms showed no significant difference (placebo=20.5, NVK=21.2, and KSK=20.8, *P*=0.56). Ct values of patients in all three arms were analyzed again at the time of discharge and compared using Kruskal-Wallis test. On day 3, the mean Ct values were found to be significantly different among 3 arms (placebo=25.1, NVK=31.5, and KSK=33.1, *P*=0.0001). Statistically significant reduction in viral load of SARS-CoV-2 was recorded in both the Siddha treatment arms NVK and KSK compared to placebo (decaffeinated tea).

**Table 4:** Cycle threshold (Ct) values analyzed on day 0 and day 3

Ct values	Arm I: Placebo Mean (SD)	Arm II: NVK Mean (SD)	Arm III: KSK Mean (SD)	***P*** value
Day 0 (*n*=38, 37, 39)	20.5 (4.3)	21.2 (2.8)	20.8 (4.3)	0.56
Day 3 (*n*=34, 33, 35)	25.1 (3.79)	31.5 (5.11)	33.1 (4.57)	0.0001

Units: Cycle threshold

Ct values were not analyzed on days 6 and 10 as all the patients of Arm II and Arm III were discharged

### Time taken for patients to become asymptomatic

**Fig. 5:**
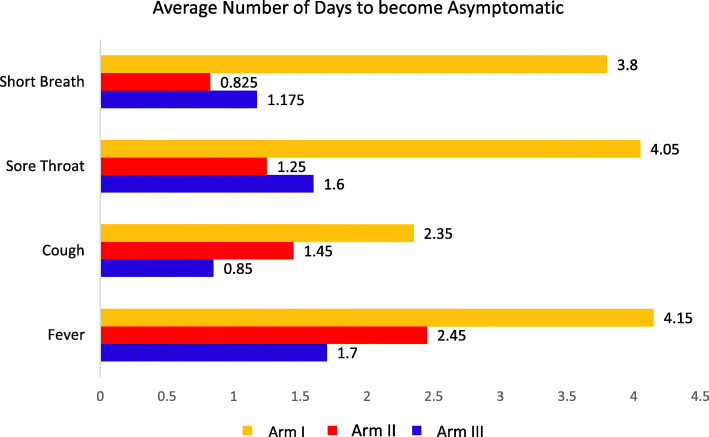
Time taken to convert patients from symptomatic to asymptomatic for Arm I: placebo, Arm II: NVK and Arm III: KSK

The average time taken for a patient to become asymptomatic from symptomatic in the standard Siddha treatment NVK and KSK arms was significantly less when compared to that taken by those in the placebo drug (decaffeinated tea) arm (Fig. 5). Time taken by patients to get asymptomatic from symptomatic were 2.5 mean days in the NVK arm; 1.7 in the KSK arm and 4.2 days in the placebo arm (Kruskal-Wallis test, *P*=0.0001). Similarly, patients in NVK arm and KSK arm took significantly lesser time (mean days) compared to placebo for both sore throat (NVK arm; 1.3, KSK arm; 1.3, placebo arm; 3.5, *P* value =0.0005) and short breath (NVK arm; 1.3, KSK; 1.3, placebo arm; 3.2, *P* value =0.0001, respectively).

### Safety evaluation

In all three groups, only two adverse events (AEs) were reported. Mild episodes of AEs of vomiting and diarrhea were observed for a single day in Arms I and II. Both the episodes were reversed within a day and treatment was continued. AEs were notified to trial site IEC and DSMB (Data Safety and Monitoring Board, Ministry of AYUSH, Govt. of India), within the reporting timelines. No serious adverse events (SAEs) were reported throughout the study.

### Mean variation in IL-6 value

One of the significant markers in COVID-19 disease is IL-6 which is indicative of immune response. During the study course, IL-6 values were recorded at baseline and on the day of discharge from the hospital (endpoint) as summarized in Table 5. Baselines IL-6 mean values were recorded as 7.5 of Arm I, 5.7 of Arm II, and 7.1 of Arm III. On the day of discharge (endpoint), IL6 values were showed a significant difference (Arm I 4.0, Arm II 2.6, and Arm III 2.2 and Kruskal-Wallis test, *P*=0.02) from baseline. This revealed an overall improvement in the IL-6 scores.

**Table 5:** Biomarker parameters recorded at time of admission (baseline) and at time of discharge from hospital (endpoint)

	Arm I: Placebo Mean (SD)	Arm II: NVK Mean (SD)	Arm III: KSK Mean (SD)	***P*** value
IL 6 baseline (*n*=38, 39, 34)	7.5 (4.4)	5.7 (4.2)	7.1 (4.0)	0.09
IL 6 endpoint (*n*=38, 39, 38)	**4.0 (2.9)**	**2.6 (2.5)**	**2.2 (1.3)**	**0.02**
LDH baseline (*n*=26, 25, 22)	17.2 (3.5)	16.6 (4.6)	17.3 (5.8)	0.61
LDH endpoint (*n*=24, 17, 13)	17.4 (5.6)	17.3 (3.9)	13.2 (5.6)	0.10
Ferritin baseline (*n*=26, 25, 21)	10.5 (4.5)	11.7 (5.3)	13.2 (5.8)	0.25
Ferritin endpoint (*n*=23, 16, 11)	11.4 (5.0)	11.0 (5.7)	10.6 (5.3)	0.95
D dimer baseline (*n*=26, 21, 13)	0.9 (0.7)	1.9 (3.6)	2.7 (6.8)	0.79
D dimer endpoint (*n*=16, 15, 7)	2.6 (4.8)	1.6 (2.6)	3.7 (7.5)	0.53
CRP baseline (n=24, 25, 22)	3.5 (2.8)	3.0 (2.4)	2.6 (1.5)	0.84
CRP endpoint (*n*=22, 19, 15)	3.9 (3.2)	2.5 (1.6)	3.1 (4.7)	0.26

**Units**: *IL 6,* picogram per milliter (pg/mL); *LDH*, units per liter (U/L); *Ferritin*, micrograms per liter (mg/L); *D*-*dimer*, nanograms per milliliter (ng/mL); *CRP*, milligrams per decilitre (mg/dL)

### Mean variation in biomarker parameters

To evaluate the overall improvement across the three arms, the other biomarker parameters considered for evaluation were LDH, ferritin, D-dimer, and CRP. These parameters were recorded at the time of admission (baseline) and at the time of discharge from the hospital (endpoint) as summarized in Table 5. The total mean value of LDH was 17 at baseline and 16.4 at the endpoint. The total mean values of ferritin, D-dimer, and CRP were 11.7, 1.6, and 3.6 at baseline showing an overall improvement at the endpoint with the mean values of 11.1, 2.4, and 3.2, respectively. The overall change in CRP, LDH, ferritin, and D-dimer was found to be non-significant (*P*>0.05) amongst all 3 arms.

## Conclusion

This is the first randomized controlled clinical trial to study the effectiveness of two classical Siddha herbal formulations, NVK and KSK, along with the standard allopathy treatment for COVID-19. Patients of NVK (Arm II) and KSK (Arm III) recovered faster than patients of placebo (Arm I) and spent fewer days in the hospital than those in the placebo arm (Arm I). All patients of both NVK and KSK arms were discharged by day 6 whereas maximum patients of the placebo arm were discharged only on day 10. Similarly, RT-PCR test was negative by day 6 in both Arms II and III, whereas for the placebo group, 61% were RT-PCR-positive. Additionally, patients of Arms II and III took significantly less time to become asymptomatic compared to the placebo arm. Between the Siddha treatments, the KSK arm showed more promising results than the NVK arm, as over 50% patients were discharged and found RT-PCR-negative even on day 3. Patients of KSK spent the least time in the hospital among all 3 arms. IL6 markers of Siddha treatment arms showed a statistically significant difference in comparison to the placebo arm. No SAEs were recorded throughout the study. The results of this trial suggest that NVK and KSK are safe and effective drugs in the management of mild to moderate COVID-19 disease when taken along with allopathy treatment.

In spite of the limited sample size, the effects of Siddha decoctions, both NVK and KSK, along with the standard of care allopathy compared to placebo have been confirmed. The effects of these drugs were also statistically significant and proved the efficacy of an integrative approach with allopathy for COVID-19 management. This trial also complies with the National Health Policy 2017 of integrative approaches of allopathy with traditional systems of medicines especially with Siddha medicines. The result of this trial encourages the integration of Siddha medicines with allopathy in combating pandemics like COVID-19 and also in repurposing existing Siddha drugs. A large-scale, multi-centric clinical trial can help to make it robust and reproducible.

## Data Availability

All participants’ data will be kept confidential and personal identifiers of the study participants will not be disclosed to the public. Only the investigators will have access to the trial data. All the procedures will be carried out by strictly adhering to the Good Clinical Practices (GCP). The monitors will have access to the study documents.

## Abbreviations

AEs: Adverse events
ARDS: Acute respiratory distress syndrome
BA: Bronchial asthma
CCRSs: Central Council for Research in Siddha
CKD: Chronic kidney disease
CRP: C-reactive protein
CTRI: Clinical Trial Registry of India
COVID 19: Coronavirus disease
CT value: Cycle threshold value
DM: Diabetes mellitus
DSMB: Data Safety and Monitoring Board
EC: Ethics committee
GCP: Good clinical practice
GIMS: Government Institute of Medical Sciences
GFR: Glomerular filtration rate
HT: Hypertension
HPTLC: High-performance thin-layer chromatography
ICU: Intensive care unit
IL6: Interleukin 6
ICMR: Indian Council for Medical Research
KSK: Kaba Sura Kudineer
LDH: Lactate dehydrogenase
LFT: Liver function test
NVK: Nilavembu Kudineer
NKA: National Kidney Association
PPVC: Peripheral Pharmacovigilance Center
RFT: Renal function test
ECG: Electrocardiogram
RT PCR: Reverse transcription-polymerase chain reaction
SD: Standard deviation
SARS COV 2: Severe acute respiratory syndrome coronavirus 2
SAE: Serious adverse events
TLC: Thin layer chromatography
OECD: Organisation for Economic Co-Operation and Development
